# Microscopic and molecular evidence of the presence of asymptomatic *Plasmodium falciparum* and *Plasmodium vivax* infections in an area with low, seasonal and unstable malaria transmission in Ethiopia

**DOI:** 10.1186/s12879-015-1070-1

**Published:** 2015-08-05

**Authors:** Lemu Golassa, Frederick N. Baliraine, Nizar Enweji, Berhanu Erko, Göte Swedberg, Abraham Aseffa

**Affiliations:** Aklilu Lemma Institute of Pathobiology, Addis Ababa University, Addis Ababa, Ethiopia; Department of Biology, LeTourneau University, Longview, Texas USA; Department of Medical Biochemistry and Microbiology, Uppsala University, Uppsala, Sweden; Armauer Hansen Research Institute, Addis Ababa, Ethiopia

**Keywords:** Asymptomatic parasitaemia, Microscopy, PCR, Rapid diagnostic tests, Unstable transmission, *Plasmodium falciparum*, *Plasmodium vivax*, Ethiopia

## Abstract

**Background:**

The presence of asymptomatic infections has serious implications for malaria elimination campaigns. Since asymptomatic carriers do not seek treatment for their infection and may become gametocyte carriers, they undoubtedly contribute to the persistence of malaria transmission in a population. The presence of asymptomatic parasitemias was noted in areas with seasonal malaria transmission. In Ethiopia there is a paucity of data regarding the prevalence of asymptomatic malaria carriage. This study was undertaken to assess the presence and prevalence of asymptomatic *Plasmodium falciparum* and *Plasmodium vivax* infections in south-central Oromia, Ethiopia.

**Methods:**

A total of 1094 apparently healthy individuals ≥ 2 years of age in south-central Oromia, Ethiopia, an area with seasonal and unstable malaria transmission, were screened for the presence of asymptomatic plasmodial infections. Finger-prick blood samples were taken from each participant for blood film preparation for microscopy and the rapid diagnostic test (RDT). Blood samples were also spotted on Whatman 3MM filter paper for parasite DNA extraction.

**Results:**

The prevalence of asymptomatic *Plasmodium* carriage (*P. falciparum*, *P. vivax* and mixed species) was 5.0 % (55/1,094) as determined by microscopy, while the prevalence as determined using RDT was 8.2 % (90/1,094). PCR was done on 47 of 55 microscopy-confirmed and on 79 of 90 RDT-confirmed samples. PCR detected parasite DNA in 89.4 % (42/47) of the microscopy-positive samples and in 77.2 % (61/79) of the RDT-positive samples. No significant difference was observed in the prevalence of asymptomatic *P. falciparum* or *P. vivax* infections in the study area (*P* > 0.1). However, the prevalence of asymptomatic parasitaemia was significantly associated with gender (OR = 0.47, P = 0.015; being higher in males than females) and age (X^2^ = 25, P < 0.001; being higher in younger than in older individuals). Age and parasite densities had an inverse relationship.

**Conclusions:**

This study confirms the presence of asymptomatic *P. falciparum* and *P. vivax* infections in south-central Oromia, an area with low, seasonal and unstable malaria transmission in Ethiopia. Of 55 microscopically confirmed asymptomatic infections, *P. falciparum* monoinfection accounted for 45.5 % and of 90 RDT positive asymptomatic infections, 66.7 % were *P. falciparum*. Although not statistically significant, *P. falciparum* accounted for a relatively large number of the asymptomatic infections as determined by both tests. The prevalence of asymptomatic parasitaemia was highest in the younger age group. HRP-2-based RDTs specific for *P. falciparum* showed high false positivity rate compared to *Plasmodium* lactate dehydrogenase (pLDH) specific to *P. vivax*. Although microscopy and RDT detected substantial numbers of asymptomatic infections in apparently healthy inhabitants, the use of a highly sensitive molecular diagnostics offers a more accurate assessment of the magnitude of asymptomatic infections.

## Background

Substantial reductions in malaria transmission and morbidity have been reported in many parts of East Africa, consequent to the spirited up-scaling of interventions in the region [1–4]. Despite this achievement, there is indication that existing interventions alone will not lead to malaria elimination in most malaria-endemic areas and additional strategies need to be considered. The human parasite reservoir consists of all malaria infections in people in a given area, including symptomatic and asymptomatic infections [5]. There has been increasing recognition that a substantial proportion of the parasite reservoir may be found in individuals who do not show symptoms and therefore do not seek health care [6]. Thus, a successful malaria elimination program calls for attention to all parasite carriers, especially asymptomatic malaria besides the diagnosis and treatment of clinical cases [7, 8]. The asymptomatic reservoir is comprised of individuals with parasites below the microscopic threshold (subpatent parasitemia) and those with parasites visible by microscopy (patent parasitemia) [8]. The WHO currently recommends malaria diagnosis either by microscopy or rapid diagnostic test (RDT) in patients with suspected malaria prior to treatment [9]. Although microscopy requires a high skill level to achieve acceptable sensitivity, it can be used for species differentiation, parasite quantification, and identification of parasite life stage. Malaria RDTs are easier to use and can detect specific *Plasmodium* parasite antigens using one or more of three target antigens: histidine-rich protein 2 (HRP2), lactate dehydrogenase (LDH), and aldolase. HRP2 is expressed only by *Plasmodium falciparum* and is the most widely used target antigen for malaria RDTs. LDH and aldolase are expressed across all *Plasmodium* species but tend to yield lower diagnostic accuracy in commercially available RDTs.

Although patients with subclinical infections do not present with malaria symptoms, they still contribute to the cycle of transmission in a population. The relative contribution of sub-clinical infections has considerable implications for the design and use of elimination diagnostics. The epidemiology of asymptomatic malaria in different transmission settings is attracting increasing attention, because asymptomatic individuals are still able to produce gametocytes and therefore provide the reservoir for onward transmission [10–14]. The presence of asymptomatic infections is less known in settings with marked seasonality [15]. However the presence of such cases has also been reported from low endemic areas such as Amazon region of Brazil and Peru [16], Colombia [17], Solomon Island [12] and Principe [18] in recent years. Evidently, any successful malaria elimination strategy will hinge on the ability to find and treat the asymptomatic reservoir.

Given the importance of asymptomatic malaria infection in different endemic areas of the world, various studies have assessed the prevalence of asymptomatic infections in the control and elimination phase of malaria, because detection and treatment of all sources of infection is very critical at this stage [7, 9, 19–22]. Ethiopia includes regions of differing malaria endemicity and transmission. In Ethiopia, multispecies rapid diagnostic tests (RDTs) are used at health posts and malaria microscopy is carried out at district-level health centers and regional-level hospitals for all suspected malaria cases. Virtually, the sources of most malaria prevalence data are usually health centers, and such reports of malaria cases restricted to people seeking treatment for their illness. Such data may represent a fraction of a population whose infection with *Plasmodium per se* would end up with clinical malaria. The objective of this study was to determine the prevalence of asymptomatic *Plasmodium* spp. infection using microscopy and RDT in a population-based cross-sectional survey in south-central Oromia, Ethiopia. PCR was not conducted on all microscopy and RDT positive samples but a subset of these. The fact that some of the microscopy and RDT positive individuals were anaemic we were unable collects blood spots for molecular methods and hence not included in the PCR analysis. It is anticipated that data from this study will have important practical implications to the malaria elimination strategies in Ethiopia.

## Methods

### Study area and population

This study was conducted in West Arsi Zone, Oromia Region, Ethiopia, located approximately at a distance of 251 km from the capital city, Addis Ababa. Malaria transmission is seasonal and unstable in this area. A cross-sectional study was conducted in 12 *kebeles* (the smallest administrative unit) of the Shalla District from November through December 2012. The *kebeles* have known population sizes and systematically registered households. Each *kebele* is sub-divided into villages. After obtaining informed consent from parents/guardians, all members of the randomly selected households were requested to give finger-prick blood samples. Inclusion criteria were that all volunteers must (1) be apparently healthy (defined as individuals with no complaints related to malaria symptoms), and (2) residing in the village for more than one year, and (3) consent to giving a blood sample. Members of a household who had contracted malaria and were treated within the previous month and/or were unwilling to participate in the study were excluded. Individuals with body temperature ≥37.5 °C as measured by digital thermometer were also excluded.

**Fig. 1:**
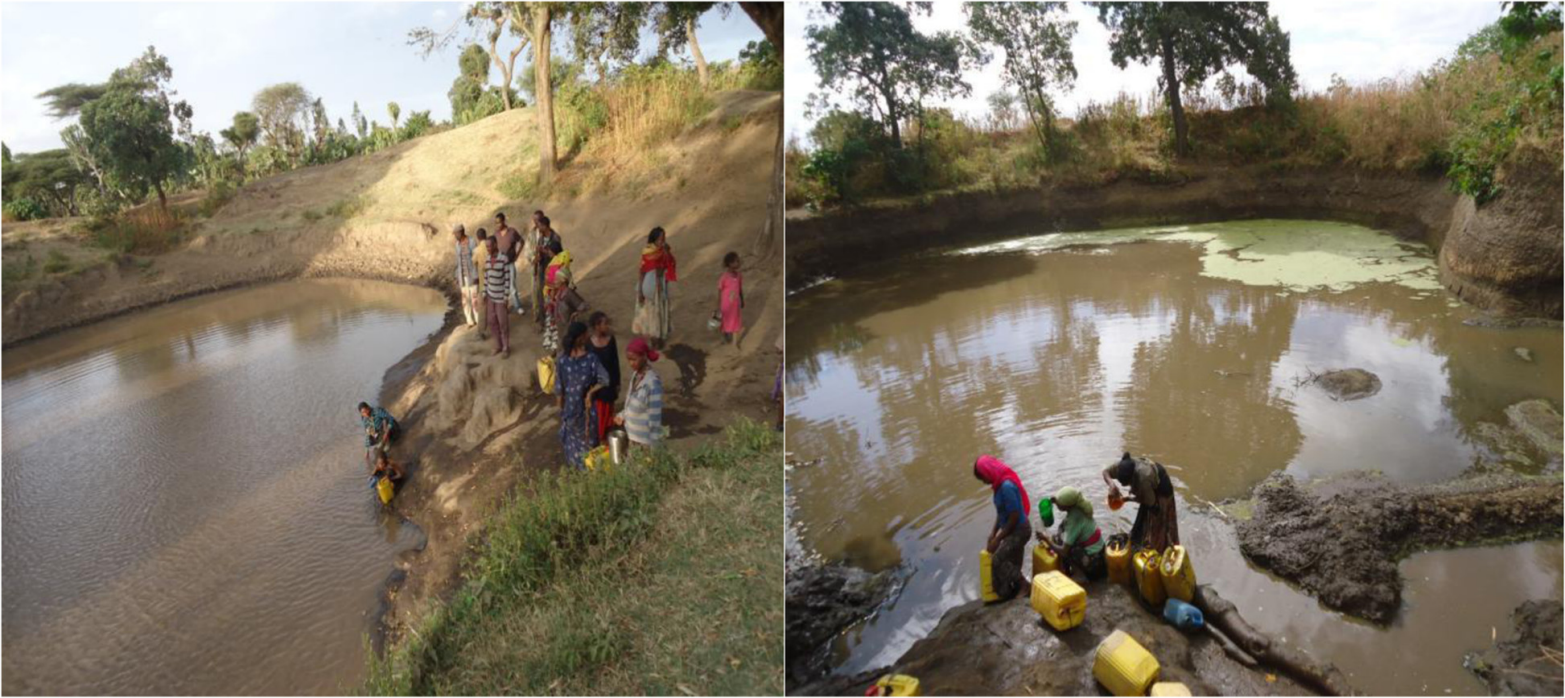
Man-made reservoir grounds to store rain water in the study area

The study area has unique feature, there is a severe shortage of surface water bodies. To overcome shortage of water bodies in the surrounding, inhabitants dig ground and use it as a reservoir to store rain water. This man-made reservoir ground dries out after a few months of the termination of the rain seasons (Fig. 1).

### Sample collection

A community-based cross-sectional study was conducted to determine the prevalence of asymptomatic *Plasmodium* spp. infections. Using 95 % confidence level, a design effect of 1.5, a margin of error of 4 %, and anticipated asymptomatic prevalence of 50 %, a total of 993 individuals were screened (using the formula N = (Zα/2)2P (1-P)*DEFF/ME2 where N is the sample size, Zα/2 is the critical α level, P is the anticipated asymptomatic malaria prevalence, DEFF is the design effect and ME is the marginal error). But to include 993 individuals in the sample, it was proposed that 1094 individuals were invited to participate, thus allowing for a 10 % non-response. Finger-prick blood samples were used for microscopy and RDT tests. For PCR analysis, blood samples were spotted onto Whatman 3MM filter paper. The filter papers were dried and stored individually in sealed plastic bags. Examination of the blood films was performed by experienced malaria microscopists. Participants tested malaria positive by RDT were treated according to the national treatment guideline: Artemether-lumefantrine for *P. falciparum* and chloroquine for *P. vivax* infected patients.

### Blood film examination and determination of parasitaemia

Thick and thin blood smears were made on the same slide, air dried and transported to the Adama Malaria Control Center. The slides were then stained with 10 % Giemsa for 15 minutes and screened for the presence of plasmodial infections. Two experienced microscopists read the slides blinded to individual’s RDT results and to each other. Fortunately, the agreement of the two microscopists was 100 % in species identification. Parasite density was determined from thick blood smears by counting the number of asexual parasites per 200 WBCs and calculated assuming a standard mean white blood cell count of 8,000 leukocytes per μl of blood [23]. A smear was considered negative if no parasites were seen after review of 100 high-powered fields. Later on, parasite densities were calculated and converted into the number of parasites per μl of blood.

### Rapid Diagnostic Tests (RDTs)

The SD BIOLINE Malaria Ag P.f/P.v POCT test kit (Standard diagnostic, Inc, Germany, Lot No. 145021) was used to capture malaria antigens and was performed in accordance with the manufacturer’s instructions. The kit targeted malaria antigens HRP-2 specific for *P. falciparum* and *Plasmodium* lactate dehydrogenase (pLDH) specific for *P. vivax*.

### DNA extraction and PCR amplification

Parasite DNA was extracted using the Chelex method [24]. PCR was done for samples tested positive by microscopy and/or RDT. The different *Plasmodium* species were identified by microscopy and species-specific nested-PCR [25]. Briefly, 3 mm^2^ piece of filter paper with blood spot was cut with an automatic cutter and placed in 1 ml of phosphate buffered saline containing 0.5 % saponin and incubated overnight at 4 °C. The brown solution was removed and replaced with 1 x PBS and then incubated for 1 h at 4 °C. The solution was removed and 150 μl of DNAse free water was added followed by 50 μl of 20 % Chelex. The tubes were placed into a heated block (99 °C) for 10 mins and vortexed every two min. This was repeated 2–3 times. The solution was centrifuged at 13, 000 rpm for 10 min. The supernatant was carefully separated and centrifuged as before. The supernatant which contains DNA was collected carefully into 0.5 ml clean tube and used for PCR immediately or stored at −20 °C until use. The extracted DNA was amplified by nested-PCR (Bioer LifePro thermal cycler). Primer sequences (5’-3’) used for the primary PCR in *P. falciparum* (outer): CCGTTAATAATAAATACACGCAG and CGGATGTTACAAAACTATAGTTACC (reverse). For the nested PCR (outer): TGTGCTCATGTGTTTAAACTT and CAAAACTATAGTTACCAATTTTG (reverse) primer sequences were used. For *P. vivax*, primers for primary PCR (outer): CGCCATTATAGCCCTGAGCA and TCTCACGTCGATGAGGGACT (reverse). For the secondary PCR (outer): GGATAGTCATGCCCCAGGATTG and CATCAACTTCCCGGCGTAGC (reverse). Primary and nested PCR assays were performed in a 20 μl volume reaction mixture of DreamTaq buffer, 100 μM dNTPs, 0.05 μM of each primers and 1.25 units of Dream-Taq enzyme (Thermo Scientific). Two microliters (2 μl) of the sample DNA were used for outer amplification. DNA from *P. falciparum* strain 3D7 and sterile water were used as positive and negative controls, respectively. For *P. vivax*, microscopy and RDT known samples were used as positive control. Amplicons were resolved in ethidium bromide-stained 1.5 % agarose gel and observed under ultraviolet transillumination.

### Ethical considerations

This protocol was reviewed and approved by the Institutional Review Boards (IRBs) of Aklilu Lemma Institute of Pathobiology, Addis Ababa University and of the Armauer Hansen Research Institute, as well as by the National Research Ethics Review Committee (NRERC). Information about the objective of the study was given to the head of each household. Written informed consent was obtained from all adults willing to participate in the study, with a parent/guardian giving consent for children. Malaria positive subjects were treated as per the national treatment guideline.

### Statistical analysis

Data were checked for completeness and consistency, and double entered into a SPSS17.0 (SPSS Inc. Chicago, USA) database. Descriptive statistical analysis was done using STATA 11.0 (STATA Corp. Texas, USA). Fisher’s exact test was used to assess the difference in the prevalence of asymptomatic infections between variable of interest. The agreements between microscopy and RDT data were assessed using Cohen’s kappa coefficient (*K*), assuming microscopy as a ‘gold standard’. Bivariate and multivariate logistic regressions were performed to estimate the association between the presence of asymptomatic *Plasmodium* carriages and variables of interest. An error probability (*P-* value) of <0.05 was considered as statistically significant.

## Results

A total of 1, 094 individuals (519 males and 575 females) from 317 households were surveyed in 12 randomly selected villages/*kebeles*. Infections were considered as asymptomatic in this study when individual’s body temperature was < 37.5 °C at presentation (and no history of fever within the last 72 hours) with microscopically and/or RDT confirmed *Plasmodium* infection. By microscopy, the prevalence of asymptomatic parasitaemia (*P. falciparum, P. vivax,* and mixed species) was 5.0 % (55/1094). Out of a total of 55 microscopy-positive samples, 45.5 % (25/55) were *P. falciparum*, 40 % (22/55) were *P. vivax* and 14.5 % (8/55) mixed (*P. falciparum* and *P. vivax*) infections (Table 1). The prevalence of asymptomatic parasitaemia was significantly associated with gender (OR = 0.47, P = 0.015); being higher in males (6.7 %) than females (3.5 %) (Table 2). The two-tailed Fisher’s exact test also showed, the difference between the prevalence of plasmodial infections between the two genders to be statistically significant (P = 0.0178). As age increased, asymptomatic parasitaemia carriage rates decreased (X^2^ = 25, P <0.001). Children below 5 years had the highest (12.7 %) whereas over 35 year-old adults had the lowest infection rates (2.4 %) as determined by microscopy.

**Table 1:** Asymptomatic *P. falciparum* and *P. vivax* infections as determined by microscopy and RDT

	RDT results				
Microscopy results	*P. falciparum*	*P. vivax*	P.f-P.v	Negative	Total
*P. falciparum*	22*	0	0	3	25
*P. vivax*	0	18*	1	3	22
P. f-P. v	0	0	6*	2	8
Negative	38	5	0	996*	1,039
Total	60	23	7	1,004	1,094

*Number of positive or negative samples detected by both microscopy and RDT

**Table 2:** Bivariate and multivariate logistic regression analysis on presence of asymptomatic carriage rates as diagnosed by microscopy in regards to age and parasite carriage associations

		Bivariate analysis		Multivariate analysis	
Characteristics	No. infected/total examined	COR (95 % CI)	*P-value*	AOR (95 % CI)	*P-value*
Sex					
Male	35/519	1.00	-	reference	reference
Female	20/575	0.49 (0.28-0.87)	0.015	0.54 (0.30-0.96)	0.036
Age in years					
≤5	21/166	1.00	-	reference	reference
6-15	18/373	0.35 (0.18-0.67)	0.002	0.35 (0.18-0.67)	0.002
16-25	5/165	0.20 (0.07-0.50)	0.001	0.22 (0.08-0.57)	0.002
26-35	8/263	0.21 (0.08-0.54)	0.001	0.24 (0.09-0.61)	0.003
>35	4/127	0.16 (0.04-0.57)	0.004	0.15 (0.04-0.54)	0.003

COR (crude odd ratio), AOR (adjusted odd ratio)

Using RDT, the prevalence was 8.2 % (90/1,094) and this was significantly higher than the infection prevalence (5 %; 55/1,094) detected by microscopy (P = 0.0034, McNemar’s test). Out of 90 RDT-confirmed positive subjects, 66.7 %, 25.6 % and 7.7 % were *P. falciparum*, *P. vivax* and mixed infections, respectively. RDT was positive in 85.5 % (47/55) of microscopy-positive samples. Microscopy and RDT had 95.2 % agreement (Kappa = 0.6248). Considering both microscopy and RDT data, no significant difference was observed in the prevalence of asymptomatic *P. falciparum* or *P. vivax* infections in the study area (Fisher’s 2-tailed *P* = > 0.1).

Of 55 microscopy and 90 RDT confirmed asymptomatic *Plasmodium* spp. infections, PCR was done on 47 and 79 of the samples, respectively. PCR detected parasites DNA in 89.4 % (42/47) of the microscopy-positive samples. On the other hand, PCR detected parasites DNA in 77.2 % (61/79) of the RDT-positive samples. Thus, the true positivity rate for microscopy and RDT relative to PCR were 89.4 % and 77.2 %, respectively (Table 3). Of microscopically confirmed *P. falciparum* infections, some turned out to be *P. vixax* and mixed by PCR. Of 21 blood-films positive for *P. falciparum*, one was diagnosed as *P. vivax*, three as mixed infections and two were confirmed negative by PCR. From a total of 19 blood-film positive for *P. vivax*, PCR confirmed 12 of them as *P. vivax*. Out of 51 RDT positive for *P. falciparum* infections, 32 were confirmed as *P. falciparum* by PCR. Of 22 RDT positive *P. vivax* infections, PCR confirmed *P. vivax* in 17 of the cases. Of 15 RDT-negative subjects, PCR confirmed the presence of asymptomatic parasitaemia in 8 of them.

**Table 3:** True positivity rate of microscopy and RDT against PCR in the diagnosis of asymptomatic *P. falciparum* and *P. vivax* infections

	PCR results				
Microscopy results	*P. falciparum*	*P. vivax*	Mixed	Negative	Total
*P. falciparum*	15*	1	3	2	21
*P. vivax*	1	12*	3	3	19
Mixed	0	2	5*	0	7
Negative	20	6	1	20*	47
Total	36	21	12	25	94
RDT results					
*P. falciparum*	32**	0	4	15	51
*P. vivax*	1	17**	1	3	22
Mixed	0	0	6**	0	6
Negative	3	4	1	7**	15
Total	36	21	12	25	94

*Number positive or negative by both microscopy and PCR, **Number positive or negative by both RDT and PCR.3

**Fig. 2:**
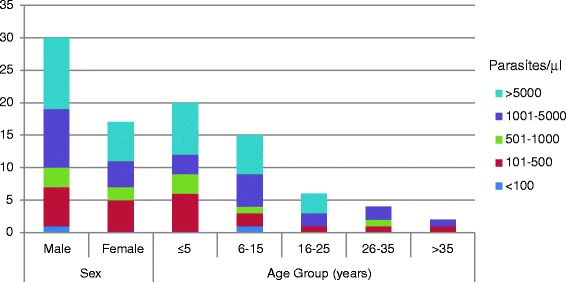
The distribution of asymptomatic *Plasmodium* spp. parasite density among the study participants with respect to age groups and sex

Age and parasite densities had an inverse relationship. Of 11 subjects with parasitaemia between 100–500 parasites per μl of blood, five *P. falciparum*, one mixed and five were *P. vivax* infection (data not shown). For parasitaemia between 500–1000 parasites per μl of blood, two were *P. falciparum* and three were mixed infection. From a total of 13 subjects with parasitaemia between 1000–5000 parasites per μl of blood, 6 *P. falciparum* and seven were *P. vivax* infections. Of 17 individuals with parasite density greater than 5000 parasites per μl of blood, four *P. falciparum*, nine *P. vivax* and four were mixed infections. The distribution of parasite densities with respect to age groups and sex is shown in Fig. 2.

From a total of 47 subjects whose parasitaemias were determined, 17 were *P. falciparum*, 22 were *P. vivax* and 8 were mixed infections. Of 17 asymptomatic *P. falciparum* cases, 58.8 % (10/17), 23.5 % (4/17) and 17.6 % (3/17) had asexual parasites, gametocytes and both asexual and gametocytes, respectively. From a total of 22 asymptomatic *P. vivax* cases, 86.4 % (19/22) had asexual parasites and 13.6 % (3/22) had both asexual and gametocytes. Six of eight mixed infections (75 %) had both asexual and gametocytes and 25 % had asexual parasites alone. The mean density of *P. falciparum* was 8074 parasites per μl of blood and 4821 parasites per μl of blood for *P. vivax* (data not shown).

**Fig. 3:**
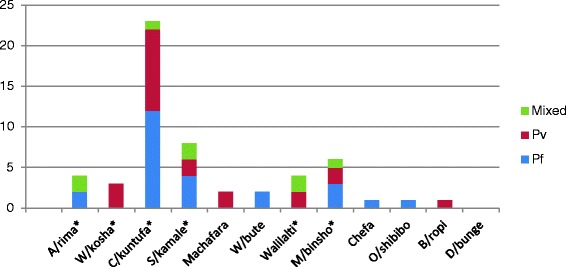
Distribution of asymptomatic *Plasmodium falciparum and P. vivax* infections in 12 study villages as determined by microscopy (N = 1094). A/rima (Algae-rima), W/kosha (Wondo-kosha), C/kuntufa, (Chiracha-kuntufa), S/kamale (Sadacha-kamale), W/bute (Waka-bute), M/binsho (Mito-binsho), O/shibibo (Ore-shibibo), B/ropi (Bilo-ropi), D/bunge (Danisa-bunge). Pf (*P. falciparum*), Pv (*P. vivax*), mixed (*P. falciparum*-*P. vivax*). In villages marked ‘*’, there is at least one man-made reservoir ground to store rain water (Fig. 1)

Asymptomatic parasitaemia was identified in 19.9 % (63/317) of the households surveyed. In W/kosha and Machafara villages only *P. vivax* was identified. In W/bute, Chefa and O/shibibo villages, only *P. falciparum* was identified. On the other hand, both *P. falciparum* and *P. vivax* were found in A/rima C/kuntufa S/kamala, Walilalti and M/binsho villages. Fig. 3 shows the distribution of asymptomatic parasitaemia in 12 study villages as determined by microscopy. Colocalisation of *P. falciparum* and *P. vivax* were noted in some of the study villages. There were many instances of detecting more than one *P. falciparum* or *P. vivax* infection within a given household. The prevalence of asymptomatic infections ranged from as low as 0 % (D/bunge) to as high as 8.5 % (A/rima) as determined by microscopy.

## Discussion

Given the renewed focus on transmission reduction leading to elimination, the quantification of the malaria parasite reservoir in a given area is important for control. In areas where malaria is sustained at low levels or is highly seasonal, asymptomatic reservoirs of infection are critical for maintaining malaria transmission [12]. Since symptomless malaria as a consequence lead to the persistence of parasite reservoirs and increases malaria transmission in human population, it can interfere with malaria elimination strategies. The present study reveals the presence of asymptomatic *P. falciparum* and *P. vivax* infections in the West Arsi Zone of the Oromia Region of Ethiopia, an area where malaria transmission is markedly seasonal and unstable. In this study, there was a slight predominance, although not statistically significant, of falciparum over vivax infections as diagnosed by both microscopy and RDT unlike in Brazil where *P. falciparum* accounted for large number of the asymptomatic infections as diagnosed by microscopy [26, 27]. The low prevalence of asymptomatic *P. falciparum* and *P. vivax* infections determined in this study is in agreement with a study in India that showed low prevalence (4.3 %) of asymptomatic *P. falciparum* and *P. vivax* infections [28].

Most children in areas with moderate-to-high levels of malaria transmission gain protection from severe disease usually by 2–5 years of age, followed by a decrease in the rate of symptomatic illness in early adolescence [29]. Given that asymptomatic parasitaemia was highest in children aged 2–5 years, malaria transmission in the study area could be higher than previously thought. We reported earlier that parasitaemias in older age groups were sub-patent that conventional malaria diagnostics, microscopy and RDT, failed to detect [30]. By adulthood, parasite densities following infection often remain at very low levels, frequently undetectable by microscopy [5] although asymptomatic parasitemia can occur at any age. The asymptomatic parasitaemia in this study was lower than the prevalence reported earlier elsewhere in Ethiopia [31]. This is because the study areas have different transmission intensities.

False positivity values were 10.6 % (5/47) for microscopy and 22.9 % (18/79) for RDT relative to PCR. Despite its many advantages, the limitations and shortcomings of microscopy are well documented [32]. The performance of microscopy depends absolutely on the quality of the equipment and reagents, the type and quality of the smear, skill of the technician, the parasite density, and the time spent on reading the smear [32]. Unfortunately, ideal conditions for effective microscopy are often not met particularly at the peripheral health care system and microscopy diagnosis could likely be prone to doubtful results. Okell et al. [33] have shown that the prevalence of infection measured by microscopy was 50.8 % of that measured by PCR and this difference is greatest in low transmission settings. Multitudes of factors are known to affect false positivity rate in RDT [32–37]. One possible limitation to our RDT data may have been that because subjects who had contracted malaria and were treated within the previous month were excluded from this study, and it was known in the community that those who would be diagnosed with infections would be freely treated, many people were enthusiastic to be part of this study and some may have concealed their previous infection history. False positive in RDT could also be due to the persistence of HRP-2 antigens post treatment [38–40].

In this study, PCR detected 20 *P. falciparum*, six *P. vivax* and one mixed infection in 47 of microscopy negative samples (that were positive by RDT), suggesting that asymptomatic parasitaemia in these samples could be below the threshold of microscopy. PCR confirmed the presence of parasite DNA in 89.4 % of microscopy-positive samples. Considering the fact that PCR can detect parasitemias as low as 0.02 parasites per μl, it is not surprising to note the superiority of the PCR over the other classical diagnostic methods [41]. PCR detected three *P. falciparum*, four *P. vivax* and one mixed infections in 15 of the RDT-negative samples, suggesting that parasite antigens in these samples could be below the detection limit of RDT. Moreover, PCR failed to detect two of the microscopy positive *P. falciparum* samples and three of the microscopy positive *P. vivax* samples. The lack of detection of infections by PCR in microscopy and RDT positive samples could be due to the small amount of DNA used in the PCR reaction as evidenced by Hodgson et al. [42] where higher sample volumes will increase the detectability of infections.

Another point worth mentioning is the missed diagnosis by microscopy and RDT observed in this study. Four of 21 (19 %) microscopy-positive *P. falciparum* samples were found to have either *P. vivax* or mixed infections. Four of 19 (21 %) microscopy-positive *P. vivax* samples were found to have either *P. falciparum* or mixed infections by PCR (Table 3). Consequently, a significant number of malaria-infected subjects were inadvertently left untreated while many parasite-free others were treated; all because of misdiagnosis by these two methods. Such diagnostic constraints of microscopy and RDT have serious implications for malaria control in the country. Studies have shown that a missed diagnosis of *falciparum* malaria increases the risk of complicated or severe disease [43, 44]. A missed diagnosis of *P. vivax* concurrent with *P. falciparum* is even more problematic since this species could cause relapses, thereby compounding morbidity. Moreover, delays in recognition and appropriate treatment of malaria increase morbidity and mortality [45]. A recent study designed to assess malaria microscopy capacity of health facilities in Ethiopia [46] showed that 51 % of the febrile patients with negative malaria laboratory test results were treated with artemether-lumefantrine or chloroquine. At present, Ethiopia has species-specific antimalarial drug regimen and therefore accurate diagnosis and prompt treatment are the key strategies to control and prevent malaria in the country. In this study, age and sex were significantly associated with asymptomatic parasitaemia carriage. Females had significantly less chance of being asymptomatic carriers compared to males. A number of studies have made the same observation and attributed it to biological and occupational differences between genders [47–50].

In line with the importance of asymptomatic infections to the infectious reservoir, the presence of gametocytes was analyzed for both *P. falciparum* and *P. vivax* infections. Of particular interest regarding the transmission potential, 41.2 % of the *P. falciparum* and 13.6 % of the *P. vivax* infections had gametocytes detected either alone or along with the asexual stages of the parasite. Moreover, gametocytes were detected in 75 % of mixed infections (Pf-Pv). The presence of asymptomatic cases with circulating sexual stages of the parasite (gametocytes) shows their potential to infect mosquitoes and cause additional human cases.

It is apparent that distance  to water is a major determinant of malaria risk. In this study, the presence of asymptomatic parasitaemia shows clustering around a few study villages. Moreover, *P. falciparum* and *P. vivax* infections were either colocalised or unique to certain study villages. In W/kosha and Machafara villages only *P. vivax* was identified. In W/bute, Chefa and O/shibibo villages, only *P. falciparum* was identified. On the other hand, both *P. falciparum* and *P. vivax* were found in A/rima C/kuntufa S/kamala, Walilalti and M/binsho villages. As to why *P. falciparum* and *P. vivax* co-exist or are unique to certain study villages needs further study. However, factors  like variations in water bodies (artificially man-made reservoir grounds for holding rain water) around the study villages may have accounted for clustering of asymptomatic infections. Indeed, geographic clustering of symptomatic and asymptomatic malaria has been reported elsewhere [11, 51]. In the study area, there is severe shortage of ground water bodies and inhabitants dig reservoirs (Fig. 1) to hold rain water and this favors vector breading.

Overall, the 5 % prevalence of asymptomatic parasitaemia as determined by microscopy in this study was substantially lower than that reported from other African countries. The prevalence of asymptomatic parasitaemia as determined by microscopy was 12 % in Gabon [52], 12.6 % in Kenya [48], 39.2 % in Mozambique [3], 35 % in Senegal [53] and 32 % in The Gambia [54]. This is not surprising because malaria transmission intensity is low in Ethiopia compared to those African countries.

## Conclusions

The main conclusions of this study were that asymptomatic *P. falciparum* and *P. vivax* infections do exist in the study area. As determined by microscopy, *P. falciparum* accounted for 45.5 % of asymptomatic infections, *P. vivax* 40 % and mixed infections 14.5 %. Asymptomatic parasitaemias were high at younger age groups, and men are at a greater risk of having asymptomatic *Plasmodium* spp. infections than women. The transmission of *Plasmodium* spp. from humans to mosquitoes requires the presence of infectious gametocytes in the human peripheral blood. Thus, the detection of asymptomatic cases with circulating gametocytes signals their importance in maintaining sustained transmission in the study area. Indeed, the presence of asymptomatic cases is a big challenge for the management of elimination programs in any malaria endemic area. As a result of a missed diagnosis by RDT on field condition, some *P. vivax* patients were treated as *P. falciparum* and vice versa as evidenced by PCR. As malaria control efforts are gaining momentum and elimination agenda is initiated, further research is needed to better understand the epidemiology of asymptomatic reservoir in Ethiopia. Although microscopy and RDT detected considerable numbers of asymptomatic infections in apparently healthy individuals, the use of a highly sensitive molecular diagnostics in large epidemiology studies offers a more accurate assessment of the magnitude of asymptomatic infections. Further studies are needed for a better understanding of the asymptomatic *Plasmodium* spp. infections and their contribution to the dynamics of malaria transmission and to the incidence of symptomatic infections.
